# The Role of the ‘Femur First’ Technique and Spinopelvic Characteristics in Achieving the Combined Sagittal Index in Total Hip Arthroplasty: Results from a Retrospective Tertiary-Center Clinical Study

**DOI:** 10.3390/jcm14165620

**Published:** 2025-08-08

**Authors:** Edoardo Guazzoni, Giuseppe Anzillotti, Francesco La Camera, Emanuela Morenghi, Guido Grappiolo, Mattia Loppini

**Affiliations:** 1 IRCCS Humanitas Research Hospital, Via Manzoni 56, 20089 Rozzano, Milan, Italyfrancesco.lacamera@humanitas.it (F.L.C.); emanuela.morenghi@humanitas.it (E.M.); guido.grappiolo@mac.com (G.G.); mattia.loppini@hunimed.eu (M.L.); 2 Department of Biomedical Sciences, Humanitas University, Via Rita Levi Montalcini 4, 20072 Pieve Emanuele, Milan, Italy

**Keywords:** combined anteversion, hip–spine, instability, pelvic tilt, total hip arthroplasty

## Abstract

**Background:** Emerging parameters, such as the spino-pelvic unit and the combined sagittal index (CSI), are gaining importance in evaluating and optimizing implant positioning in total hip arthroplasty (THA). Our institution adopts the “femur first” technique to achieve the desired combined anteversion (CA). The purpose of this study is to evaluate the role of the ‘femur first’ technique and spinopelvic characteristics in achieving the CSI_standing_ ‘safe zone’ in primary THA. **Methods:** Consecutive patients undergoing primary THA were included in the present retrospective study. All patients underwent radiographic assessments in the standing position with the EOS 2D/3D radiography system. **Results:** Forty patients (40 hips) were enrolled. Of these, 34 patients fell inside the CSI_standing_ “safe zone” (205–245°). When considering the restricted CSI_standing_ “safe zone” for patients at high risk for adverse spinopelvic characteristics (215–245°), only 16 patients fell inside the range. We demonstrated a positive linear correlation between CSI_standing_ and CA (*p* < 0.0001). Pelvic tilt (PT) showed a positive correlation both in standing and relaxed sitting positions, (*p* < 0.001). Sacral slope (SS) showed a significant positive correlation in the relaxed sitting (*p* = 0.003) position but not in the standing position (*p* = 0.34). The correlation analysis between CSI_relaxed-sitting_ and ΔSS showed a positive correlation (*p* = 0.003). **Conclusions:** The “femur first” technique is able to achieve the CSI “safe zone” in most patients; however, it seems insufficient in those with adverse spinopelvic characteristics, who are at higher risk of dislocation. Moreover, the CA, the position of the pelvis in space (PT), and its mobility (ΔSS) greatly influence the CSI “safe zone” in patients undergoing primary THA.

## 1. Introduction

Deserving the title of ‘operation of the century’, total hip arthroplasty (THA) has revolutionized the life of patients suffering from hip osteoarthritis (OA), and it is expected that the total number of procedures performed will grow up to 635,000 by 2030 [1,2,3]. As a direct consequence, the number of revision procedures is in constant increase, reaching 50.000 yearly only in the US with related costs up to $1 billion [4,5,6,7]. When evaluating causes of revisions, recurrent dislocation appears to be the most represented; thus, new parameters are emerging to evaluate and properly perform the implant position [8,9,10]. Among these, the spino-pelvic unit and the resulting hip–spine relationship are becoming particularly relevant in preoperative planning [11,12,13,14,15]. Heckman et al. introduced a new parameter to assess the sagittal plane of movement, the combined sagittal index (CSI), which is the sum of Pelvic Femoral Angle (PFA) and Anteinclination (AI) (CSI = PFA + AI). This parameter can be calculated in both the standing and relaxed-seated position and is a predictor of impingement and dislocation risk [16]. Heckman et al. introduced a CSI_relaxed-sitting_ “safe zone” of CSI_relaxed-sitting_ > 158.5° for risk for anterior impingement and posterior dislocation [14]. Grammatopoulos et al. introduced a “safe zone” for this parameter. A CSI_standing_ under 215° could expose the patients to posterior instability and a CSI_standing_ over 245° could expose the patients to anterior instability. Hence, the suggested window of security is 205° < CSI_standing_ < 245° for patients without spinopelvic pathology and 215° < CSI_standing_ < 235° in case of unbalanced or rigid spine. Values beyond these ranges have been considered to be at increased risk of dislocation [17].

Our institution has so far adopted a ‘Femur First’ Technique to obtain the desired combined anteversion (CA), translating in a proper orientation of both acetabular and femoral components. It represents a smart method able to achieve a satisfactory implant positioning in a non-navigated and non-robotic way, through a simple though reproducible surgical technique [18]. However, no studies were conducted to evaluate the eventual relation between this surgical technique and the emerging spinopelvic characteristics. Hence, the purpose of the present study is to evaluate the role of the ‘Femur First’ Technique, CA, and spinopelvic characteristics in achieving the CSI ‘safe zone’ in primary THA.

Our primary endpoint was to evaluate if patients undergoing THA with the “femur first” technique would fall inside the CSI “safe zone”.

Our secondary endpoints were correlation between the CSI_standing_ value and the CA value; correlation between the CSI_standing_ value and the femoral torsion (FT) value; correlation between the CSI_standing_ value and the pelvic tilt (PT) or the sacral slope (SS) in the standing position; correlation between the CSI_relaxed-sitting_ value and the PT or SS in the sitting position; and correlation between the CSI_relaxed-sitting_ value and spine mobility.

## 2. Materials and Methods

### 2.1. Study Population

All individual participants signed written informed consents for undergoing the surgery and for inclusion in the registry of orthopaedic surgical procedures, within the scope of research and improvement of clinical practice. The study was conducted in accordance with the Declaration of Helsinki and good clinical practice guidelines. The study protocol was approved by the Ethics Committee of IRCCS Humanitas Research Hospital (protocol code 618/17). Consecutive patients undergoing primary THA were included in the present study. Inclusion criteria included patients >18 years old eligible for primary THA with diagnosis of primary OA or secondary OA due to mild development dysplasia, Perthes disease, slipped capital femoral epiphysis, and post-trauma osteoarthritis. Exclusion criteria were patients eligible for partial or total THA revision, THA associated with other procedures (i.e., femoral osteotomy), previous pelvic and/or femoral osteotomy, severe hip dysplasia (Crowe III or IV), primitive or metastatic tumors of hip joint, previous spine and/or sacroiliac joint instrumentation, previous or current hip joint infection, and previous hip surgery of any type. All patients underwent the same surgical technique. Briefly, in lateral decubitus, after the neck osteotomy femoral rasps were inserted up to the desired size, which is left in situ. After the exposure of the acetabulum and a sequential reaming, the femur was then reduced with the proper sized rasp in situ by using a 44 mm trial plastic head with a long neck. After reduction, with the hip in neutral position at 0° of flexion and abduction, the femur was internally rotated to reach an angle of 35°, the value of the mean combined anteversion suggested by most authors, between the longitudinal axis of the tibia and the operating table [19]. Only cementless short-stem femoral components were used across all cases. Since the CA is the degree of internal rotation to produce a coplanar head and cup, the definitive cup was placed parallel to the horizontal line on the trial head in both the axial and coronal planes [18].

### 2.2. Radiographic Assessment

**Figure 1:**
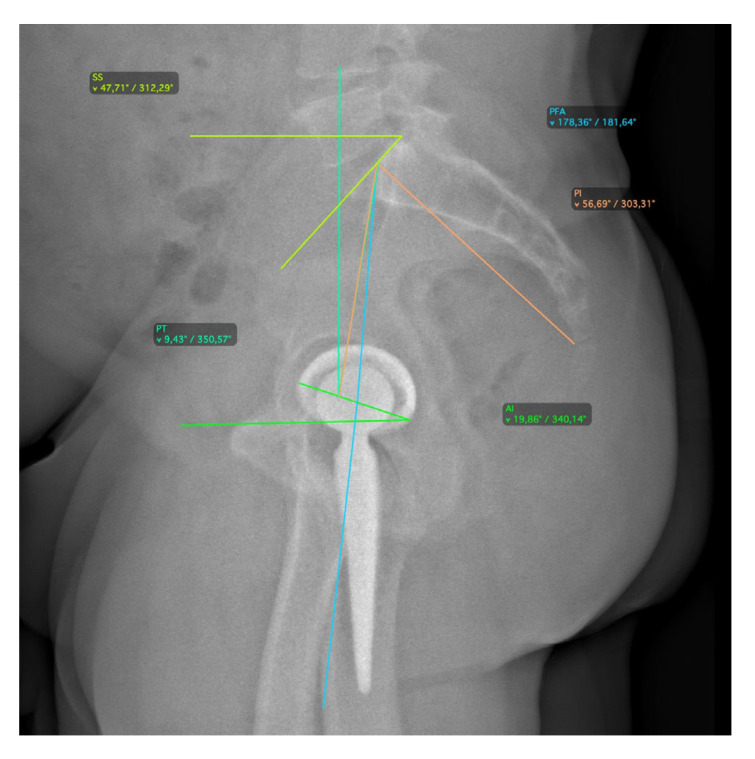
Radiographic measurements for spinopelvic parameters in standing position.

**Figure 2:**
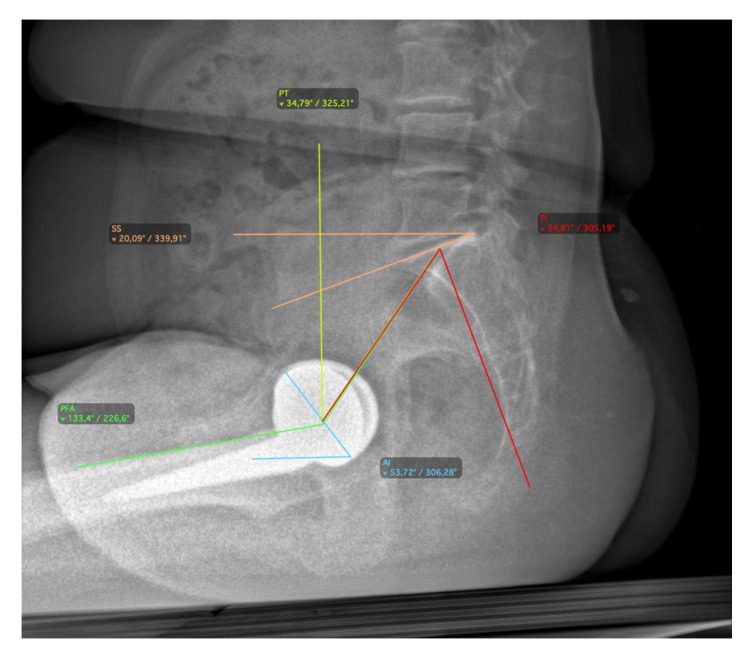
Radiographic measurements for spinopelvic parameters in relaxed-sitting position.

All patients underwent a radiographic assessment in a standing position with the EOS 2D/3D radiography system (Biospace Med, Paris, France), performed preoperatively and at 3 months follow-up, as per department usual practice. The EOS system allows one to achieve an antero-posterior (AP) and lateral radiographic view of the whole skeletal system [20,21,22]. The 2D images were used to perform a 3D reconstruction of the skeletal system and prosthetic components with dedicated software (sterEOS 3D, version 1.5.3.7947, Biospace Med, Paris, France). In addition, 3D images were used to preoperatively measure the caput-collum-diaphyseal (CCD) angle and femoral antetorsion [23], postoperative acetabular abduction (AAb) and anteversion (AA), and postoperative FT [24]. The combined anteversion was determined by the following formula: cup anteversion + (0.7 × stem antetorsion) [25]. The AAb and AA were measured in the patient frame based on a vertical plane passing through the center of the acetabular cup, which avoids the effect of a potential axial rotation of the pelvis during acquisition. On the other hand, the FT was measured relative to posterior bi-condylar plane. The CSI is the algebraic sum of PFA and AI (CSI = PFA + AI). This parameter can be calculated both in the standing and relaxed sitting position. The PT was measured on a lateral X-ray of the pelvis as the angle between a line drawn from the midpoint of the bicoxofemoral axis to the midpoint of the sacral plate and the vertical [26]. The SS were measured on a lateral X-ray of the pelvis as the angle between the superior plate of S1 and a horizontal line to the floor [27]. Spine mobility was defined by the difference in SS from the standing and relaxed sitting position (ΔSS). Spinopelvic mobility was classified based on ΔSS as previously described [14,27]: stiff (<10°), normal (10–30°), and hypermobile (>30°). A ΔSS < 5° was considered suggestive of a biologically or surgically fused spine [28]. The AI was measured on a lateral X-ray of the pelvis as the angle between a line tangent to the anterior and posterior edges of the cup and a horizontal line parallel to the margin of the radiograph [29]. The PFA was measured on a lateral X-ray as the angle subtended by a line connecting the midpoint of the S1 end plate and the center of the bicoxofemoral axis and a line from the center of the bicoxofemoral axis projected distally toward the center of the knee, typically along the anterior shaft of the proximal femur [28] (Figure 1 and Figure 2).

### 2.3. Data Analyses

All the analyses were performed using Stata for Windows (version 18.0, StataCorp LCC, College Station, TX, USA). Descriptive statistics were calculated. The categorical variables were expressed as a frequency with percentage. Continuous variable data were expressed as a mean with standard deviation and range as minimum and maximum values. Correlation analysis was performed among the considered radiological indices. The *p* was considered significant for values <0.05.

## 3. Results

Forty patients (40 hips) undergoing primary THA were enrolled. Seventeen patients were men. The mean age was 61 years (36–84) at the time of the index procedure. Mean BMI at the surgery was 27 (18–39). The preoperative diagnosis was primary osteoarthritis in 33 patients, osteoarthritis secondary to mild development dysplasia of the hip in 6 patients, and post-trauma osteoarthritis in one patient. ΔSS was calculated for all patients and spinopelvic mobility was categorized accordingly (Table 1).

**Figure 3:**
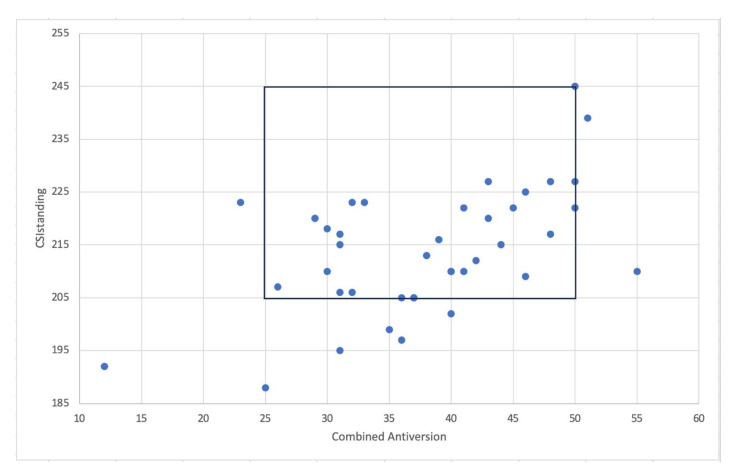
The graph shows the patients inside both combined anteversion “safe zone” and CSI_standing_ “safe zone”.

All patients underwent THA through the “femur first” technique to reach the optimal intraoperative combined anteversion. Of these, 34 patients (85%) fell inside the CSI_standing_ “safe zone” (205–245°) (Figure 3).

**Figure 4:**
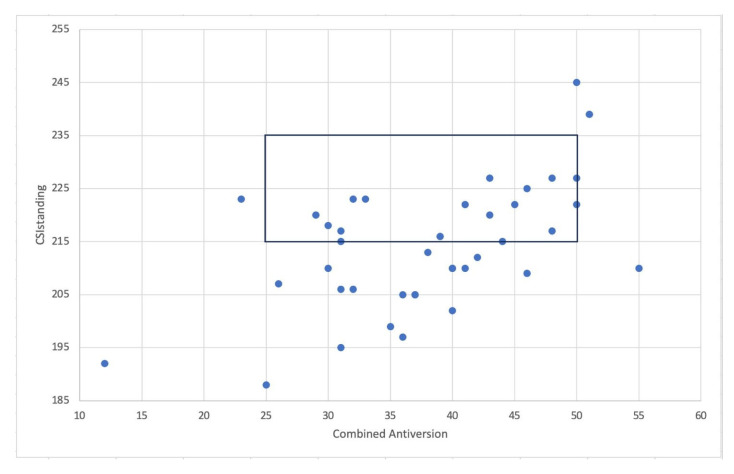
The graph shows the patients inside both combined anteversion “safe zone” and CSI_standing_ “safe zone” for patients with adverse spinopelvic characteristics.

When considering the restricted CSI_standing_ “safe zone” for patients at high risk for adverse spinopelvic characteristics (215–245°), only 16 (40%) patients fell inside the range (Figure 4).

**Figure 5:**
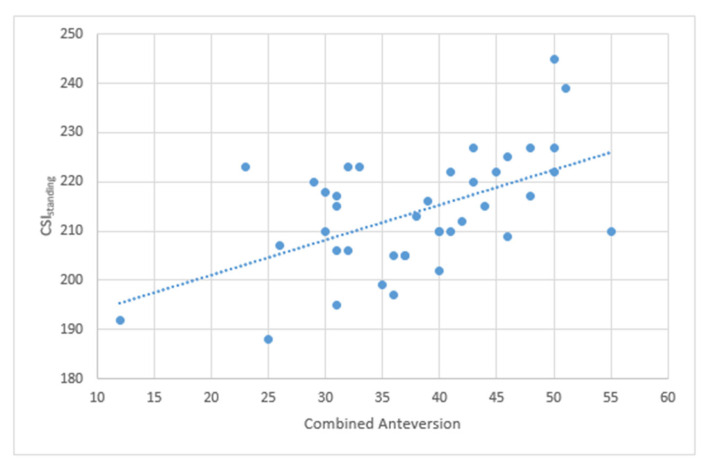
The graph shows the positive linear correlation between CA and CSI_standing_.

A further correlation analysis was performed, and it demonstrated a linear regression between combined anteversion value and the CSI_standing_ value. The correlation coefficient was r = 0.54, and the regression analysis showed a statistically significant correlation (r = 0.54, coefficient 0.71, 95%CI0.35; 107, *p* < 0.0001) (Figure 5).

The subgroup analysis showed no statistically significant difference when adjusting the correlation for sex (*p* = 0.37) and age (*p* = 0.51).

**Figure 6:**
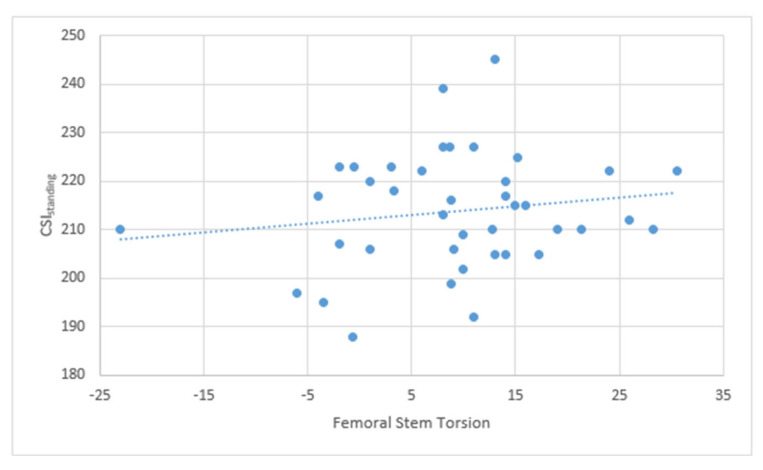
The graph shows the positive linear correlation between FT and CSI_standing_.

The correlation analysis between CSI_standing_ and FT showed a positive correlation r = 0.18, though not statistically significant (Figure 6).

The study on the positional parameters of the pelvis and the CSI_standing_ or CSI_relaxed-sitting_ showed a statistically significant positive correlation for most of the parameter in either position. PT showed a significant positive correlation both in a standing (r = 0.6548, coefficient 1.07, 95%CI 0.67; 1.47, *p* < 0.001) and in a relaxed sitting position (r = 0.6360, coefficient 1.27, 95%CI 0.76; 1.78, *p* < 0.001). SS showed a significant positive correlation in a relaxed sitting (r = −0.4572, coefficient −0.75, 95%CI −1.23; −0.27, *p* = 0.003) position but not in a standing position (r= 0.25, 95%CI −0.27, 0.78, *p* = 0.34).

**Figure 7:**
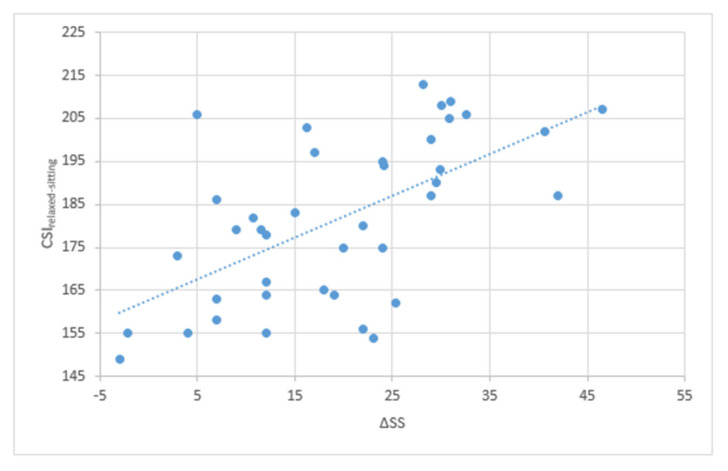
The graph shows the positive linear correlation between ΔSS and CSI_relaxed-sitting_.

The correlation analysis between CSI_relaxed-sitting_ and ΔSS showed a statistically significant positive correlation with a correlation coefficient of 0.82 (*p* = 0.003) (Figure 7).

## 4. Discussion

The main finding of the present retrospective clinical study is that the “femur first” technique is able to achieve the CSI “safe zone” in most patients; however, it seems insufficient in those with adverse spinopelvic characteristics, who are at higher risk of dislocation. Moreover, the CA, the position of the pelvis in the space (PT), and its mobility (ΔSS) greatly influence the CSI “safe zone” in patients undergoing primary THA.

The continuous effort in terms of dislocation prevention is given by the current trends in hip arthroplasty surgery. In fact, despite these efforts, the revision rate for dislocation in the last 8 years has not diminished significantly. This is demonstrated by the annual report from the British NHS, which shows a slight decrease in the overall revision rate from 1 per 1000 THA to 0.76 per 1000 THA. However, the revision rate in the first year after surgery has actually increased, from 2.33 per 1000 THA to 2.46 per 1000 THA [30]. Hence, the last few years have witnessed the rise of the functional and personalized positioning of the acetabular and femoral component to reduce the postoperative dislocation rate [11]. The supporters of this new approach claim that placing the cup and the femoral stem with functional or personalized technique should prevent impingement, whether it be implant on implant, implant on bone, or bone on bone, in each position that the patient is able to achieve [31]. The advent and spread of navigation systems and robot-assisted surgery, enabling surgeons to position the components with minimal error, have paved a new era in the field of THA. These innovations allow the operator to aim for very precise target zones, usually required in functional or personalized implant positioning [32]. However, to date there is not unanimous agreement on what is the “correct positioning” of the implants, and which is the correct “safe zone” that should be aimed for. CSI_Standing_ “safe zone” is a promising target that considers the sagittal component positioning and the sagittal balance of the patient. In 2021, Grammatopoulos et al. defined two CSI_standing_ “safe zones”: one for patients without adverse spinopelvic characteristics (205° < CSI_standing_ < 245°) and one for patients with adverse spinopelvic characteristics (215° < CSI_standing_ < 235°), defined by the authors as unbalanced (PI-LL > 10°) and/or rigid (ΔSS < 10°) lumbar spine [17]. In the present study, it was demonstrated that performing a THA with a “femur first” technique can result in having 85% of patients (34 patients out of 40) falling inside the CSI_standing_ safe zone of 205–245. It is known that having an implant outside this “safe zone” increases your risk of dislocation (odds ratio [OR]: 4.2; 95% confidence interval [CI]: 2.2 to 8.2; *p* < 0.001). When considering the “safe zone” for patients at high risk for their spinopelvic characteristics, only 40% (16 patients out of 40) fell inside the CSI_standing_ safe zone (215–245°). Being outside the “safe zone” exposes these patients to an even greater risk of dislocation (OR: 5.1; 95% CI: 1.8 to 14.9; *p* = 0.001) [17].

Falling inside the CSI_standing_ 205–245° “safe zone” four out of five times is an interesting result, considering that this specific “safe zone” was not aimed for. It is possible that these results were retrieved thanks to this specific surgical technique, which aims at achieving a perfect coplanarity between the implants, not only regarding the CA value but also the abduction angle of the cup. Furthermore, extreme anatomical abnormalities were corrected, such as high-grade ante- or retroversion of the femur or the acetabulum.

This hypothesis is further strengthened by the statistically significant linear positive correlation between the CA value and the CSI_standing_ with an increase of 0.71° of CSI every 1° of CA value. This could be interpreted as a significant finding that advances our understanding of how different parameters and “safe zones” are interconnected.

When evaluating the restricted CSI_standing_ 215–235° “safe zone”, only 40% (16 out of 40) of the patients fell inside the target. These results could be considered unsatisfactory, as patients who need to achieve this restricted “safe zone” are those at higher risk of dislocation. Therefore, it is obvious that the current “femur first” technique needs to be improved to better target both the CA and the CSI_standing_ “safe zone”. To achieve such a narrow target, the use of a navigation system or robot-assisted surgery might be required. Moreover, it is predictable that, once these new technologies prove their efficacy and reliability, they could be adopted for all patients, regardless of their risk factors.

Moreover, a positive correlation between the FT and the CSI_standing_ value was found, with a coefficient of 0.18 (for every degree of anteversion of the femoral stem the CSI increase of 0.18°), but it was not statistically significant. This is possibly related to the small sample of this study, and it should be tested on a larger sample size. The low value of the coefficient is probably a consequence of acetabular anteversion being one of the three parameters forming the AI (acetabular anteversion, angle of abduction of the cup, and pelvic tilt) [29]. These findings support the idea that correcting extreme abnormalities in femoral version helps to achieve the CSI_standing_ “safe zone”. A similar conclusion was drawn by Loppini et al., advising against positioning the acetabular cup in severe anteversion or retroversion, to compensate for femoral abnormalities [18]. In this study it was demonstrated that positional parameter of the pelvis, such as PT and SS, and mobility of the pelvis, such as ΔSS, influence greatly the CSI_standing_ and CSI_relaxed-sitting_. PT has a statistically significant linear positive correlation with CSI_standing_ and CSI_relaxed-sitting_. SS has a statistical correlation with CSI_relaxed-sitting_ but not with CSI_standing_. The authors believe this could be attributed to the limited sample size of the study. This is because the positional parameters of the pelvis are governed by a mathematical formula (Pl = PT + SS) and exhibit an inverse relationship [33]. Therefore, it is necessary to conduct further testing with a larger sample size. Furthermore, it was observed that these correlations are analogous to the femoral anteversion value, as Al is also influenced by PT and SS. Additionally, a statistically significant positive linear correlation between CSl_relaxed-siting_ and ∆SS was established, from the standing to the sitting position, with a correlation coefficient of 0.82. ΔSS represents the mobility of the spine necessary for the acetabulum to open up when transitioning from the standing to the sitting position [34]. Patients with low ΔSS have the tendency of having a low CSI_relaxed-sitting_ value, exposing them to the risk of anterior impingement and posterior dislocation when transitioning to the sitting position. While this relationship was previously recognized in the literature, to our knowledge, this is the first time it was demonstrated in clinical practice [35].

Nonetheless, the present study is not free from limitations. A formal power calculation was not performed due to the retrospective nature and limited patient sample, hence limiting the generalizability of our findings. In fact, while several statistically significant associations were identified, suggesting an adequate statistical signal, these findings should be interpreted with caution and validated in future prospective studies with predefined sample size estimation. Additionally, although a consistent surgical approach and implant system were used, unmeasured variables such as lumbar degenerative disease, spinopelvic stiffness, or patient-reported outcome scores may have influenced implant positioning or outcomes and should be investigated in future studies. Moreover, the retrospective nature of the study, the absence of a control group, and the impossibility to categorize the patients for their spinal characteristics due to the lack of the lumbar spine in the available X-rays, further reduce the strength of the retrieved results. Additionally, although a consistent surgical approach and implant system were used for all patients, we acknowledge that not measured variables, such as undiagnosed lumbar spine stiffness or other spinal disease, may influence pelvic dynamics and implant orientation. These potential confounders should be explored in future prospective studies that include dedicated lumbar imaging and spinal mobility assessments. These limitations highlight the need for further investigations to eventually confirm our hypothesis.

## 5. Conclusions

The “femur first” technique is able to achieve the CSI “safe zone” in most patients; however, it seems insufficient in those with adverse spinopelvic characteristics, who are at higher risk of dislocation. Moreover, the CA, the position of the pelvis in space (PT), and its mobility (ΔSS) greatly influence the CSI “safe zone” in patients undergoing primary THA.

## Figures and Tables

**Table 1:** The table shows the distribution of the ΔSS across the patients.

Category	Value	Number of Patients
Biological/Surgically fused	ΔSS < 5°	5
Stiff	5° < ΔSS < 10°	4
Normal	11° < ΔSS < 29°	22
Hypermobile	ΔSS > 30°	9

## Data Availability

The data supporting the reported results can be found in a repository (Zenodo).

## Abbreviations

The following abbreviations are used in this manuscript:

PFA	pelvic femoral angle (PFA)
OA	osteoarthritis
AI	anteinclination
CSI	combined sagittal index
THA	total hip arthroplasty
CA	combined anteversion
CCD	caput-collum-diaphyseal
AA	anteversion
FT	femoral torsion
AP	antero-posterior
AAb	acetabular abduction
PT	pelvic tilt
SS	sacral slope
